# Contact networks structured by sex underpin sex‐specific epidemiology of infection

**DOI:** 10.1111/ele.12898

**Published:** 2017-12-20

**Authors:** Matthew J. Silk, Nicola L. Weber, Lucy C. Steward, David J. Hodgson, Mike Boots, Darren P. Croft, Richard J. Delahay, Robbie A. McDonald

**Affiliations:** ^1^ Environment and Sustainability Institute University of Exeter Penryn TR10 9FE UK; ^2^ Centre for Ecology and Conservation University of Exeter Penryn TR10 9FE UK; ^3^ Department of Integrative Biology University of California, Berkeley 3040 Valley Life Sciences Building Berkeley CA 94720 USA; ^4^ Centre for Research in Animal Behaviour University of Exeter Exeter EX4 4QG UK; ^5^ National Wildlife Management Centre, Animal and Plant Health Agency Woodchester Park Nympsfield, Stonehouse GL10 3UJ UK

**Keywords:** Badger, bovine tuberculosis, epidemiology, exponential random graph model, multilayer network, reproductive behaviour, Social structure, zoonotic disease

## Abstract

Contact networks are fundamental to the transmission of infection and host sex often affects the acquisition and progression of infection. However, the epidemiological impacts of sex‐related variation in animal contact networks have rarely been investigated. We test the hypothesis that sex‐biases in infection are related to variation in multilayer contact networks structured by sex in a population of European badgers *Meles meles* naturally infected with *Mycobacterium bovis*. Our key results are that male‐male and between‐sex networks are structured at broader spatial scales than female‐female networks and that in male‐male and between‐sex contact networks, but not female‐female networks, there is a significant relationship between infection and contacts with individuals in other groups. These sex differences in social behaviour may underpin male‐biased acquisition of infection and may result in males being responsible for more between‐group transmission. This highlights the importance of sex‐related variation in host behaviour when managing animal diseases.

## Introduction

The importance of host social structure to infectious disease dynamics is increasingly well understood in both human (Newman 2002; Danon *et al*. 2011) and non‐human animals (Hamede *et al*. 2009; Cross *et al*. 2012; Weber *et al*. 2013b). Real‐world social networks are typically non‐random and heterogeneous (Eames & Keeling 2002; Newman 2002; Volz & Meyers 2007; Read *et al*. 2008; Danon *et al*. 2011) and incorporating such social structure into epidemiological models, rather than assuming random mixing of the population, can have important implications for predictions about the spread of infection at the level of the individual (VanderWaal & Ezenwa 2016b; Silk *et al*. 2017a; White *et al*. 2017) and of the population (Jacquez *et al*. 1988; Eames & Keeling 2002; Newman 2002; Volz & Meyers 2007). This information will be especially beneficial to understanding epidemiological patterns and to informing disease management, particularly if it can be linked to other host traits (VanderWaal & Ezenwa 2016b; Silk *et al*. 2017a).

In many systems, social structures vary with respect to qualitatively distinct types of interaction (Blyton *et al*. 2014). This results in groups, classes or categories of hosts that differ in their social interactions, with potentially important epidemiological implications (VanderWaal & Ezenwa 2016b; Silk *et al*. 2017a; White *et al*. 2017). Differences in social interactions between the sexes are likely to be particularly significant in this regard. There is widespread evidence of sex‐related variation in the epidemiology of animal diseases (Guerra‐Silveira & Abad‐Franch 2013; McDonald *et al*. 2014) and differences in the networks of same‐sex and between‐sex contacts may therefore be a key driver of this variation. In wild animals, within‐ and between‐sex contact networks will vary across the annual cycle, due to seasonal differences in reproductive and territorial behaviours. Seasonal variation in territoriality is especially likely to influence within‐sex and between‐sex contact networks, while variation in reproductive activity might have greater effects on between‐sex networks, as shown for Tasmanian devils (*Sarcophilus harrisii*) (Hamede *et al*. 2009). Despite the likely general importance for epidemiology of sex‐related variation in social contacts, empirical determination of this variation and the analytical means to relate such network dynamics to infections in natural populations have so far been lacking. Resolving between‐sex variation in contact network structures and relating this to the epidemiology of infection therefore requires an analytical framework in which detailed longitudinal information about social behaviour and infection can be integrated.

We have investigated the dynamics of within‐ and between‐sex contact networks in a wild population of European badgers (*Meles meles*) naturally infected with *Mycobacterium bovis*, the causative agent of bovine tuberculosis in cattle. Badger populations represent a significant reservoir of *M. bovis* infection in the UK and Ireland (Griffin *et al*. 2005; Godfray *et al*. 2013; McDonald 2014) and have been the subject of various management interventions to attempt to control disease in cattle. Badger social structure varies considerably across the species’ range but in large parts of the UK badger populations consist of relatively stable territorial groups, sharing communal dens, known as setts (Roper 2010). In such high‐density populations, male and female badgers differ in seasonal patterns of social behaviour (Weber *et al*. 2013a), including in behaviours, such as aggressive interactions, that are implicated in the transmission of *M. bovis* (Jenkins *et al*. 2012). The sexes also differ in their rates of acquisition and progression of infection and associated mortality, such that males, when compared to females, are more likely to become infected and then experience accelerated progression to disease and augmented mortality (Graham *et al*. 2013; McDonald *et al*. 2014). Finally, it has been shown that in a static contact network, badgers testing positive for *M. bovis* infection tended to occupy network positions with more between‐group and fewer within‐group social contacts (Weber *et al*. 2013b). This system is, therefore, ideal for testing the hypothesis that sex biases in infection are related to sex differences in host contact patterns.

The development of social network analysis has enabled the quantification of social structure in a number of species (Hamede *et al*. 2009; Stehlé *et al*. 2011; Weber *et al*. 2013b; Blyton *et al*. 2014). Social network analysis uses graph theory to quantify the complex and dynamic networks that often typify social systems, identifying patterns that scale from individual behaviour through to population structure (Grueter *et al*. 2012; Pinter‐Wollman *et al*. 2014; White *et al*. 2017). Together with the increased availability and affordability of proximity loggers that automatically record patterns of social contact (Cross *et al*. 2012; Krause *et al*. 2013; Barrat *et al*. 2014), these approaches can facilitate novel analyses of the role of social contact networks in the dynamics of infections (VanderWaal & Ezenwa 2016b; Silk *et al*. 2017a,2017b; White *et al*. 2017). One such opportunity is the application of multilayer network analysis (Kivelä *et al*. 2014; Pilosof *et al*. 2017) that can help distinguish between qualitatively different types of social interactions or dynamic processes and has been used successfully in theoretical models (De Domenico *et al*. 2016) but has not so far been widely applied to empirical data.

We have deployed a novel, multilayer network analytical approach to reveal the sex‐related dynamics of contact network structures in badgers and to test how these relate to *M. bovis* infection. Our approach combined exponential random graph models (ERGMs) with community‐based network analyses, to quantify differences in the structure of empirically determined male‐male, female‐female and between‐sex contact networks of badgers in the long‐term study population at Woodchester Park, Gloucestershire, UK (Delahay *et al*. 2013; Weber *et al*. 2013b). First, we investigated seasonal variation in how well the structure of each of these networks compared to a) social groups determined by the optimization of network modularity, and b) spatial groups determined by usage of setts. This comparison makes it possible to identify differences in the spatial scale over which contacts are structured, for within‐ and between‐sex networks. We predicted that social groups would tend to explain the likelihood and strength of contacts better than spatial groups, but that this would vary among different types of contact and be seasonally variable. Specifically, we expected the greatest changes, especially for contacts among males, in spring, when territorial and reproductive behaviour is most expressed. We then examined how between‐group contacts (defined using social and spatial methods) at an individual‐level related to infection status for each of the three sex‐structured networks, predicting that there would be a positive correlation between infection status and the proportion of between‐group contacts, but expecting that this may vary among the three networks. In this way, we provide a rare empirical investigation of the importance of sex differences in social behaviour and their association with sex biases in the spread and persistence of infection.

## Methods

### Study system

We used proximity‐logging radio tags (Sirtrack, Havelock North, New Zealand) mounted on leather‐collars deployed on 51 individual badgers (24 males and 27 females) in a population at Woodchester Park. This population has been the subject of long‐term ecological and epidemiological research, in which badgers are trapped at all active setts four times a year. All badgers are marked with a unique tattoo when first captured, and at every subsequent capture they are anaesthetized and measures of condition are made alongside collection of a series of samples to determine their infection status (McDonald *et al*. 2018). Data were collected between June 2009 and May 2010. At this time, the total population of adults and sub‐adults was between 134 and 201 individuals (Weber *et al*. 2013b), living in 20 territories identified by bait‐marking (Delahay *et al*. 2000a). Network data were collected for individuals captured at eight territories in the centre of the study population. The 51 collared badgers represent *c*. 68% of the badgers captured in the eight territories during this period. Although this represents only a sub‐sample of the population, and animals living at the edge of the sampled study area will have more network connections than recorded, numerous between‐group contacts were still available.

Proximity logging devices were individually set to begin recording a proximity event (hereafter termed contact) when two or more animals came within 0.64 ± 0.04 m of one another (Weber *et al*. 2013a). The end of a contact was recorded after the animals had been out of detection range for 30 seconds or more. At this point the date, time and duration of the contact, and identity of the individuals contacted was logged by the device. The short‐range detection distance used was chosen to be within the likely transmission distance for aerosols of *M. bovis*. Contact data were downloaded whenever collared badgers were recaptured.

### Social network construction

The study period was split into four seasons; summer (June–August), autumn (September–November), winter (December–February) and spring (March–May). All individuals for which collars were known to be active for at least 30 days of a given season were included in that season's networks. Prior to analysis, all contacts recorded during trapping operations and 36 hours after the release of the animal were removed. Networks were then constructed separately for male‐male, female‐female and between‐sex contacts. For this study, contact networks were weighted by the total duration of contacts rather than the frequency of contacts. Individuals that were present but did not form within‐ or between‐sex contacts, and therefore did not form part of that network within a season were deleted from that network type for that season. The resulting size and density of each of these networks, as well as their mean degree and mean strength, in each season are presented in Table 1. Network density is provided as the number of social contacts that have occurred, as a fraction of the total number of possible social contacts.

**Table 1:** Key summary statistics for badger contact networks

		Summer	Autumn	Winter	Spring
Male‐male	Nodes	20	21	22	20
	Edges	35/190	30/210	36/231	22/190
	Mean degree	3.50 (1.54)	2.86 (0.96)	3.27 (1.55)	2.2 (1.15)
	Mean strength	96 079 (58 541)	20 980 (14 526)	77 036 (101 274)	49 825 (66 544)
Female‐female	Nodes	24	23	23	19
	Edges	33/281	30/258	23/258	23/171
	Mean degree	2.75 (1.36)	2.61 (1.78)	2.00 (1.76)	2.41 (2.12)
	Mean strength	77 867 (77 389)	24 226 (25 207)	33 382 (42 232)	6974 (12 527)
Between‐sex	Nodes	44	44	45	39
	Edges	85/480	69/483	68/506	53/380
	Mean degree	3.86 (1.91)	3.14 (1.66)	3.02 (1.90)	2.72 (1.95)
	Mean strength	87 561 (74 764)	25 020 (22 862)	46 016 (52 458)	16 986 (33 762)

The number of nodes (badgers), density of edges (social contacts), mean (and standard deviation) degree and mean (SD) strength of the networks for the three different types of social interaction in each season. Edge density is given within parentheses as number of social contacts/maximum number of possible social contacts. Not all individuals recorded in each season are present in every network for that season as some individuals did not form within‐ or between‐sex contacts in that season.

### Determination of infection status

An individual was defined as test‐positive for *M. bovis* infection if it had, at any point in its capture history (prior to or during the study period), provided a positive response to one or both of two diagnostic tests: a badger‐specific lateral flow immunoassay (Stat‐Pak Brock ELISA (Chambers *et al*. 2009) or an interferon‐gamma release assay (Dalley *et al*. 2008). Combining tests in this way increases the likelihood of correctly assigning infection status and, in this population, with the two tests used in parallel on a single occasion, the positive and negative predictive values were estimated to be 0.75 and 0.97 respectively (Drewe *et al*. 2010). We acknowledge the imperfect performance of the diagnostic tests used. However, for convenience hereafter we denote test‐positive individuals as infected and test‐negative individuals as uninfected. By the end of the study period, 27 badgers were identified as infected (test‐positive) and 24 as uninfected (test‐negative).

### Community assignment

The assignment of individuals to communities was made using social contact data from the entire study period. Community assignment was completed using the multilevel community detection algorithm in igraph (Csardi & Nepusz 2006). This algorithm builds communities in a stepwise fashion, beginning by assuming that each node in the network is its own community and then (re)assigning each node to the community (another node or set of nodes) that maximizes its modularity at each step of the algorithm, until no nodes can be reassigned. It then repeats this process by attempting to merge communities obtained from the first stage of the algorithm, stopping when it is not possible to maximize modularity scores any further. For the original algorithm, see Blondel *et al*. (2008).

### Network‐level community structure

For each network type (male‐male, female‐female and between‐sex), in each season, we constructed two weighted exponential random graph models (Krivitsky 2012) using the R packages ergm (Hunter *et al*. 2008; Handcock *et al*. 2015) and ergm.count (Krivitsky 2015) to explain network structure. These models included 5 terms: (1) nonzero (to account for the zero‐inflation of edges), (2) sum (equivalent to the intercept in a generalized linear model), (3) the distance between the location of the main setts of two individuals as a dyadic edge covariate, (4) shared group membership as a dyadic edge covariate and (5) infection status (infected and uninfected) as a nodal covariate. For between‐sex networks, we additionally fitted a dyadic edge covariate identifying whether two individuals were the same sex or not (binary). By fixing the parameter value for this effect to ‐∞ it was possible to prevent the model fitting edges between individuals of the same sex. Edge weight was fitted using a Poisson reference distribution after edge values for all completed edges had been log‐transformed (as part of this transformation, values were rounded to the nearest integer and 1 was added to all values so that the minimum strength of a completed edge was 1). Model convergence was checked by visual inspection of Monte Carlo Markov Chains (MCMCs) for parameter estimates and using the function mcmc.diagnostics() in the R package ergm.count (Lusher *et al*. 2013; Silk & Fisher 2017; Silk *et al*. 2017b) after MCMCs were run for 50 000 iterations with a thinning interval of 1000. Within each pair of models, one model (hereafter called the spatial model) defined shared group membership using spatial information (sett usage) and the other (hereafter called the social model) used social information from community assignment analysis, based on contact events. For male‐male and female‐female networks, we compared the fit of alternative models using differences in Akaike's Information Criterion scores (ΔAIC), and determined that one model fitted better when ΔAIC>2. This was not possible for between‐sex networks as the inclusion of a term with a fixed parameter value prevented model AIC from being calculated. Therefore, we also used the estimate of the shared group membership term from the models to compare directly how contact strengths differed when groups were defined by either spatial or social factors. Model estimates in count ERGMs are positive when a given network configuration has greater edge weights than expected and negative when that network configuration has lower edge weights than expected. In this case, shared group membership was expected to have a positive effect on contact strength within a dyad (i.e. individuals have significantly stronger contacts with individuals from their own rather than different groups), but the magnitude of that positive model estimate provides information on how well either social groups or spatial groups captured differences in contact strengths (i.e. if the positive estimate was greater for social groups than spatial groups, then there was a bigger difference between within‐social‐group and between‐social‐group contacts than there was between within‐spatial‐group and between‐spatial‐group contacts).

### Between‐group contacts and infection status

A network community and ERGM‐based approach was used to investigate differences in the between‐group contacts of individuals, and their association with infection status in male‐male, female‐female and between‐sex networks throughout the year. For this analysis, seasonal networks for each type of contact were combined, as between‐group contacts are relatively rare events. The three combined networks were filtered to contain only between‐group contacts and these were analysed using ERGMs that contained the terms nonzero (for zero‐inflation), sum (an intercept for edge weight), distance between the main setts of two individuals (as a dyadic edge covariate) and infection status. Once again for the between‐sex network, we fitted a dyadic variate with a fixed parameter value (−∞) that prevented the model from fitting edges between individuals of the same sex. For the results presented in the main text, networks were filtered using spatial groups. Using spatial groups better represented differences in contacts that relate to the wider spatial spread of infection, and provided a measure that could be quantified in the absence of network data in other study systems. However, when the analysis was conducted using more conservative social groups the results were qualitatively similar (these results are provided in Supplementary Information). Model convergence was assessed using Markov chains after 50 000 iterations with a thinning interval of 1000, as described above. Estimates of the nonzero term enabled differences in the frequency of between‐group contacts to be compared for within‐sex networks, which were of similar size and density (but not for between‐sex networks, in which density is inherently lower). Estimates of the sum term facilitated comparisons of the strength of between‐group contacts between all networks, and estimates for the node attribute factor term for infection status on edge weight enabled the effect of infection status on contacts to be determined for each of the three types of network.

## Results

**Figure 1:**
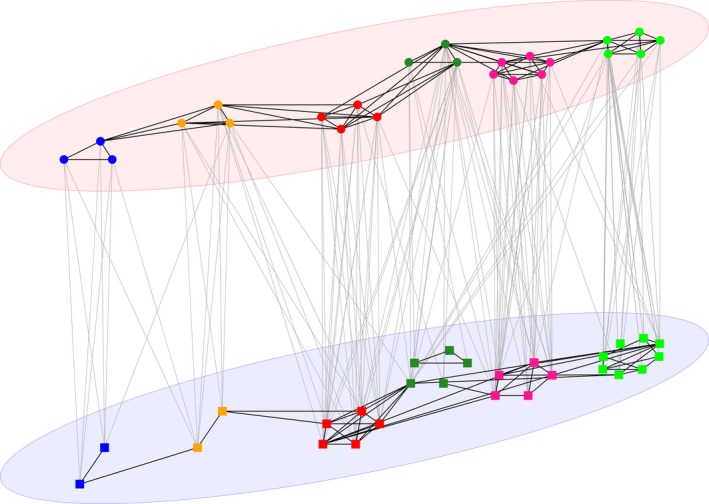
A multi‐layer social network of male (red ellipse) and female (blue ellipse) badgers. Male‐male and female‐female contacts are shown by black lines within ellipses and between‐sex contacts are shown by grey lines between layers. Node colour indicates social group membership for each individual based on the annual network.

**Figure 2:**
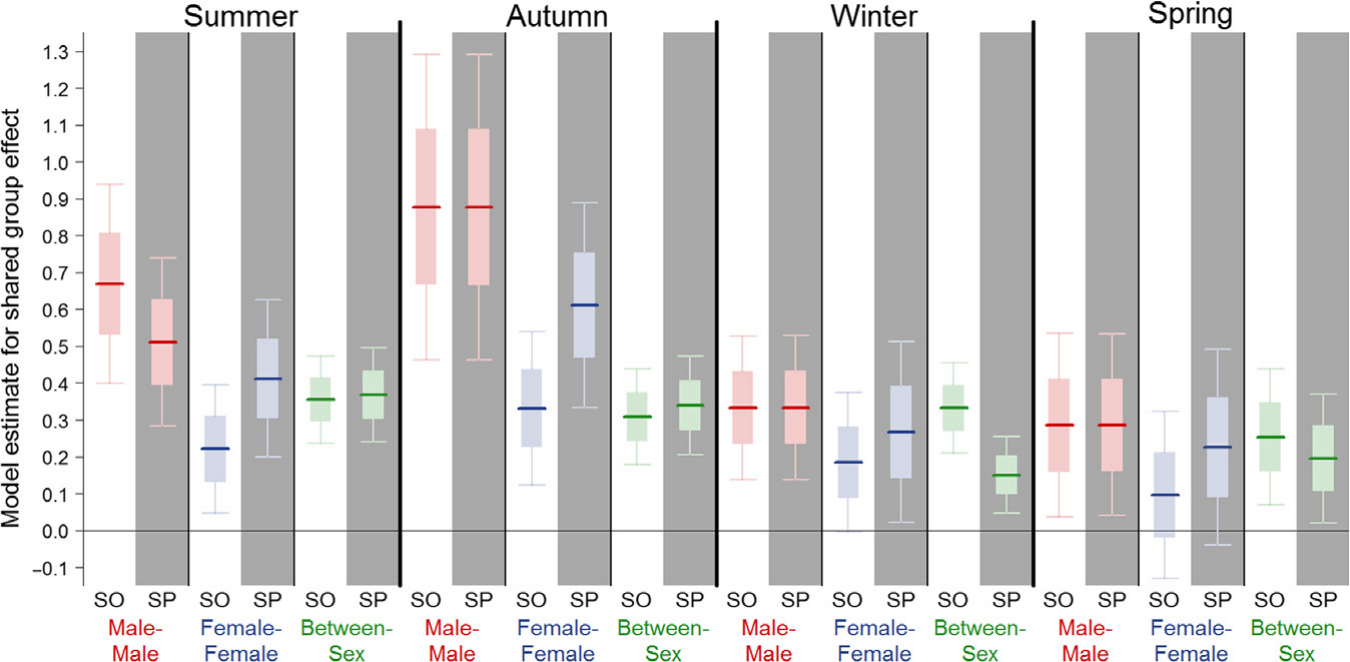
Model estimates for a shared group membership effect in exponential random graph models of social contacts of badgers by network type and season. The size of the effect represents the extent to which contacts are stronger within a spatial (SP) or social (SO) group, than outside it. Comparison of the different effects with spatial or social definitions of a group identifies changes with season in the relative strength of social contacts from between‐group to within‐group. Dark lines represent the model estimate, light boxes represent the standard error and bars represent the 95% confidence intervals.

The population‐level group structure resembled both within‐ and between‐sex social contacts over the course of the entire year of the study (Fig. 1), though there were key differences in the structuring of contacts within these networks layered by sex. For example, in one group (depicted by dark green nodes in Fig. 1), all three male individuals are connected, while two distinct cliques are apparent based purely on female‐female contacts. Male‐male and between‐sex network structures were better explained using social groups for at least part of the year, while female‐female network structure was typically much better explained using spatial groups (Fig. 2, Table 2). There were seasonal differences in all three different types of network. For male‐male contacts, the social model only had substantially lower AIC values during the summer months, whereas during other seasons, the model fits were very similar between the social and spatial models (Table 2). For between‐sex networks, the social model had a considerably stronger effect on contact strength than the spatial model during winter (Fig. 2). In contrast, there was minimal evidence for seasonal dynamics in female‐female networks and the spatial model had lower AIC values than the social model in all seasons apart from winter. During winter, the AIC values for the spatial model remained smaller but models fitted similarly well (Table 2).

**Table 2:** Seasonal variation in the relative performance of social and spatial models in describing the structure of male‐male and female‐female contact networks of badgers

Network type	Season			
	Summer	Autumn	Winter	Spring
Male–Male	**Social**>Spatial 11.42	Social≈Spatial −0.28	Social≈Spatial 0.26	Social≈Spatial 0.21
Female–Female	Social<**Spatial** −10.70	Social<**Spatial** −16.22	Social≈Spatial −0.65	Social<**Spatial** −2.11

Model performance was compared using Akaike's Information Criterion; the better performing model is shown in bold and the number given represents AIC_Spatial_‐AIC_Social_, hence a negative number indicates better fit for the Spatial model. The best‐fitting model was undetermined if ΔAIC<2 and models were considered to be similar. Model fit was compared between two equivalent exponential random graph models where shared group membership was defined either using social groups, based on community structure derived from contact events, or spatial groups, based on main sett affiliations. Between‐sex networks are not included as AIC measures could not be calculated (see Methods).

The results based on model fits were strongly supported by differences in contact strengths identified from the model estimates of the shared group membership effect (Fig. 2). In each case where model fit suggested that either the spatial or social model was more appropriate, the magnitude of the shared group membership effect for that model was correspondingly greater, indicating a more evident transition in contact strength from between‐group to within‐group contacts. For male‐male networks, the estimate of the shared group effect was considerably higher for the social model rather than the spatial model during the summer, representing a tendency for the difference in strength seen in within‐group and between‐group contacts to be much higher when groups were identified using social contacts rather than spatial sett usage. However, in other seasons estimates were almost identical. For between‐sex networks, the shared group estimates were higher in the social model than spatial model for all seasons, with the difference being greatest in winter. For female‐female networks, the shared group estimates were consistently higher for the spatial model, representing much reduced contact strengths as soon as individuals were not present at the same sett. In accord with the results obtained from overall model fit, the effect was strongest in autumn and smallest in winter.

### Individual contacts and infection status

**Figure 3:**
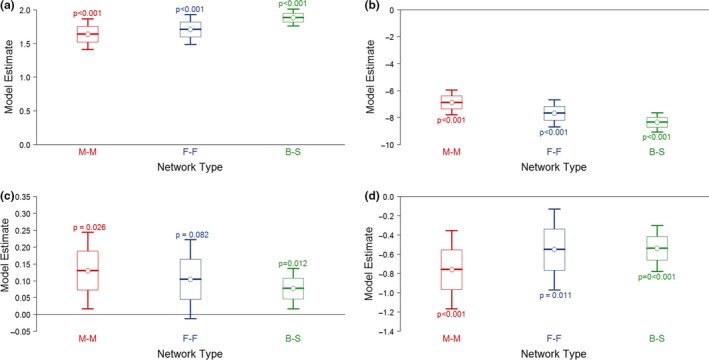
Model estimates from an exponential random graph model of between‐group social contacts in badgers for (a) the strength of contacts (sum term), (b) the degree of contacts (nonzero term), (c) the positive effect of TB infection status on strength (node factor term) and (d) the effect of distance between main setts on strength (edge covariate term). Groups are defined spatially using the sett at which badgers were caught. A comparable figure from a similar analysis based on social groups defined by community assignment is provided in the Supplementary Information. Results are shown for male‐male networks (red), female‐female networks (blue) and between‐sex networks (green). Points are the model estimate, boxes are the standard error and bars are the 95% confidence intervals around this estimate. *P* values are for the difference between that estimate and 0.

Individuals that were infected (test‐positive) tended to have more between‐spatial‐group contacts in all three network types. This effect was statistically significant for male‐male (0.130 ± 0.058, *P* = 0.026) and between‐sex (0.077 ± 0.031, *P* = 0.012) networks when spatial groups were used (Fig. 3c). If social groups were used, then statistical significance of this effect persisted only for male‐male networks (0.157 ± 0.066, *P* = 0.017; Fig. S1). There was a significant negative correlation between the distance separating main setts and the probability of contacts (male‐male: −0.760 ± 0.208, *P* < 0.001; female‐female: −0.550 ± 0.214, *P* = 0.011; between‐sex: −0.529 ± 0.122, *P* < 0.001 for spatial groups; Fig. 3d, Fig. S1). Using these models, it was possible to compare the frequency and strength of between‐group contacts in male‐male and female‐female networks, and determine the effect of distance on the strength of between‐group contacts; between‐group contacts tended to be more likely in male‐male networks (nonzero: −6.88 ± 0.48) than female‐female networks (nonzero: −7.69 ± 0.51) (Fig. 3b, Fig. S1b), although they were no stronger (sum: 1.64 ± 0.12 [male‐male] c.f. 1.71 ± 0.11 [female‐female]; Fig. 3a, Fig. S1a) and decreased markedly in frequency/strength with distance (Fig. 3d, Fig. S1d).

## Discussion

We have demonstrated clear differences in the spatio‐temporal dynamics of within‐ and between‐sex contact networks in a wild population of European badgers. Furthermore, by distinguishing between different types of social interactions, we found that differences in network structure and the relationship between individual network position and infection status, provide a potential mechanism for the apparent sex bias in favour of males acquiring and potentially spreading infection (Graham *et al*. 2013). This is important because it identifies a link, though we cannot ascribe causality, between‐sex differences in the epidemiology of infection and sex‐related variation in contact patterns. This also highlights the importance of considering variation in social interactions between the sexes when describing the dynamics of infection and disease.

Using ERGMs in a novel multilayer network analysis of variation in community structure, we have shown that male‐male and between‐sex contacts occur at a broader spatial scale than female‐female contacts and are more important in connecting the population and forming wider social networks. Our approach reveals the potential consequences of social structures at different spatial scales for infection transmission. In our case, spatial groups, defined by shared sett use, represented a smaller spatial scale than social groups, identified by analysis of the annual network of contacts. By resolving the overall contact network into an interdependent, multilayer network we were able to consider the community structure obtained from three different types of interaction and reveal the importance of male‐male and between‐sex social contacts in linking together multiple groups.

Increased structure in networks has been associated with reduced transmission of infection at both population (Salathé & Jones 2010) and group (Griffin & Nunn 2012; Nunn *et al*. 2015) levels, and individuals that connect otherwise distinct communities may represent important social bottlenecks to the spread of infection (Nunn *et al*. 2015). In moderate to high density badger populations, in which badgers live in territorial groups and where within‐group interactions are much more frequent than between‐group interactions, *M. bovis* infection tends to be both spatio‐temporally aggregated (Delahay *et al*. 2000b; Woodroffe *et al*. 2005) and correlated with the frequency of movements among main setts (Rogers *et al*. 1998; Vicente *et al*. 2007). Therefore, our results suggest that contacts involving males are likely to play a disproportionately important role in the spatial spread of infection. In heterogeneous populations, where individuals vary in their importance in spreading infection, targeting particular individuals can improve the effectiveness of management interventions (Lloyd‐Smith *et al*. 2005; Delahay *et al*. 2009; VanderWaal & Ezenwa 2016b; Silk *et al*. 2017a). As a result, knowledge that males tend to occupy more important roles in a badger contact network could facilitate the targeting of management interventions.

The positive correlation between individuals being test‐positive for *M. bovis* infection and between‐group contacts was more apparent in male‐male and between‐sex networks than in female‐female networks. Together with evidence that male social contacts tend to occur on a broader spatial scale than female contacts, this could provide a behavioural explanation for the male‐biased risk of acquiring infection in this population (Graham *et al*. 2013) and the high risk of males acquiring infection in female‐dominated groups (Vicente *et al*. 2007). This in turn could operate alongside, or interact with, the tendency for males to have reduced immuno‐competence (Guerra‐Silveira & Abad‐Franch 2013; Klein & Flanagan 2016). In badgers, the primary cell‐mediated response to *M. bovis* infection is weaker in males compared to females of similar age (Beirne *et al*. 2016), suggesting that both mechanisms could play a role. For example, it has been suggested that there may be behavioural costs associated with having more between‐group contacts and fewer within‐group contacts, such as elevated stress or reduced body condition (Vicente *et al*. 2007), and given the differences in network position these are more likely to impact males. It may also be that a combination of risky social behaviour and increased susceptibility could have a synergistic effect on the risks of becoming infected and subsequent progression of infection to disease and/or infectiousness.

More rapid progression of disease in male badgers (Graham *et al*. 2013; McDonald *et al*. 2014) could also help explain the stronger relationship between contact network position and infection status. Infected badgers make more use of outlier setts (Weber *et al*. 2013a) and tend to range further than uninfected badgers, once disease has progressed (Garnett *et al*. 2005). If progression of disease is faster in infected males than infected females, then these changes in social behaviour may be more pronounced or occur sooner after an individual is infected. Our results therefore also point towards the importance of sex differences in changes in social‐spatial behaviour subsequent to infection. We were unable to explore the directionality of the relationship between infection and social behaviour in this study, and therefore are unable to determine whether sex differences in the association between contact network position and infection is cause, consequence or a combination. Nevertheless, both alternative explanations provide some valuable insights into how such sex‐related differences in epidemiology might arise. For example, it may be that the co‐dynamics of behaviour and infection in male badgers may disproportionately contribute to the spread and maintenance of infection within populations (Funk *et al*. 2010; Ezenwa *et al*. 2016). It also raises the possibility that pathogen‐induced changes in host behaviour might have the potential to affect network modularity and thereby facilitate spread of infection (Salathé & Jones 2010; Volz *et al*. 2011). The low incidence rate (new incident cases) of *M. bovis* infection in our study population prevented us from assessing the directionality of the relationship between behaviour and infection, but this represents an important area of future research into sex bias in transmission of infection.

Finally, we have revealed variation in the seasonal dynamics of within‐ and between‐sex social contacts. Badger contact networks are shaped by a combination of spatial constraints and social behaviour. While our method cannot disentangle the relative importance of these drivers for contact network structure, it is clear that differences in contact network dynamics can be attributed to behavioural and physiological differences between male and female badgers that influence their socio‐spatial behaviour. In badgers, there is considerable seasonal variation in behaviour (Roper 2010; Weber *et al*. 2013a; Silk *et al*. 2017c), and even for animals sharing dens and territories this can result in seasonal differences in the daily patterns of contact frequency and duration (Silk *et al*. 2017c). Our multilayer analysis revealed that male‐male contact network structure was better explained by social groups during summer, when individuals are least territorial (Roper *et al*. 1986) and the use of outlier setts is relatively high (Weber *et al*. 2013a). In contrast, between‐sex networks are best explained by social groups during winter, when activity levels are reduced but levels of reproductive activity are greatest (Roper 2010). Badgers are known to mate with individuals from both their own and neighbouring territories (Carpenter *et al*. 2005; Dugdale *et al*. 2007), and therefore it seems likely that it is this reproductive activity that links assemblages of setts into broader groups during this period. In winter and spring, male‐male contacts are no better explained by social groups than spatial groups. While there is increased territoriality during this period (Roper *et al*. 1986, 1993), this could decrease contacts between animals in different territories or increase them if there are agonistic interactions on territory boundaries (Macdonald *et al*. 2004), so the role of territoriality is not immediately clear. It may be that reduced outlier sett use (Weber *et al*. 2013a) and ranging behaviour (Roper 2010) play a role during these periods. These differences in seasonal dynamics of host contact structure could have important implications for infection dynamics, for example in their relationship with sex‐specific patterns of seasonal forcing of infection (Altizer *et al*. 2006; Grassly & Fraser 2006).

By combining a community‐based approach with ERGMs in an interdependent multilayer network, we were able to generate new insights into sex differences in contact network structure, and how this was related to infection by an endemic pathogen. Taking an explicitly multilayer approach facilitated the direct comparison of community structure for within‐ and between‐sex contacts. While we could still provide useful insights by analysing each layer with a separate model, as ERGMs become more developed to deal with multilayer networks of various descriptions (e.g. Heaney 2014; Bodin *et al*. 2016), further useful insights could be provided by revealing how dependencies between the network layers shape overall network structure. For example, Bodin *et al*. (2016) have used new structural configurations within ERGMs to investigate human social‐ecological systems, and a similar approach applied to animal behaviour could identify how contact patterns generated by qualitatively different types of social interaction might contribute to key ecological processes such as infection spread.

Overall, our results reveal the importance of considering different types of social network interactions when linking social networks and infection dynamics. We found evidence that relationships between social behaviour and infection status were explained predominantly by male social behaviour, which provides a potential mechanism for enhanced infection risk in male hosts. We also highlight the key role of seasonal reproductive behaviour in driving differences between within‐ and between‐sex network dynamics, which may in turn influence seasonal forcing of infection in natural populations. Given that sex biases in infection risk have been documented in a variety of host‐pathogen systems (Guerra‐Silveira & Abad‐Franch 2013), considering the role of sex‐based network substructure may be fundamental to improving the control of disease.

## Authorship

All authors designed the study. NW collected the data. MJS and LCS analysed the data with help from DJH and DPC. MJS and RM wrote the manuscript with all authors contributing to revisions.

## Data Availability

All data and R code are available at Data Dryad: https://doi.org/10.5061/dryad.s1502
