# Supplementary figures and images for: Contact networks structured by sex underpin sex‐specific epidemiology of infection
Source: Ecol Lett. 2017 Dec 20;21(2):309–18. doi: 10.1111/ele.12898 (PMC6849844; doi:10.1111/ele.12898)

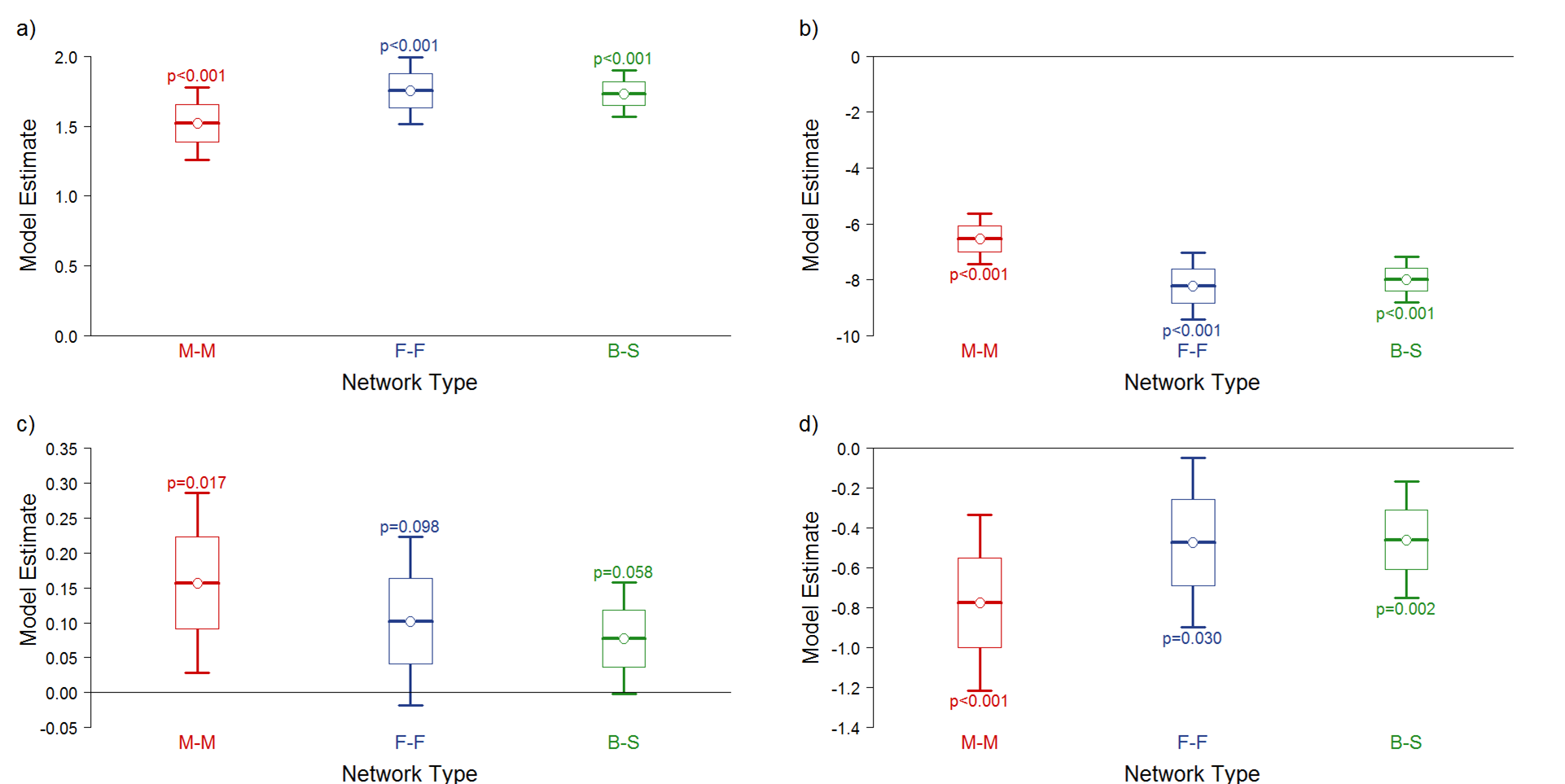

Supplement: Supplementary file 1 [file ELE-21-309-s001.tif]
