# Supplementary material for: Contact networks structured by sex underpin sex‐specific epidemiology of infection
Source: Ecol Lett. 2017 Dec 20;21(2):309–18. doi: 10.1111/ele.12898 (PMC6849844; doi:10.1111/ele.12898)
Supplement: Supplementary file 2 [file ELE-21-309-s002.docx]

**Supplementary Information**

**Contact networks structured by sex underpin sex-specific epidemiology of infection**

Matthew J. Silk, Nicola L. Weber, Lucy C. Steward, David J. Hodgson, Mike Boots, Darren P. Croft, Richard J. Delahay and Robbie A. McDonald


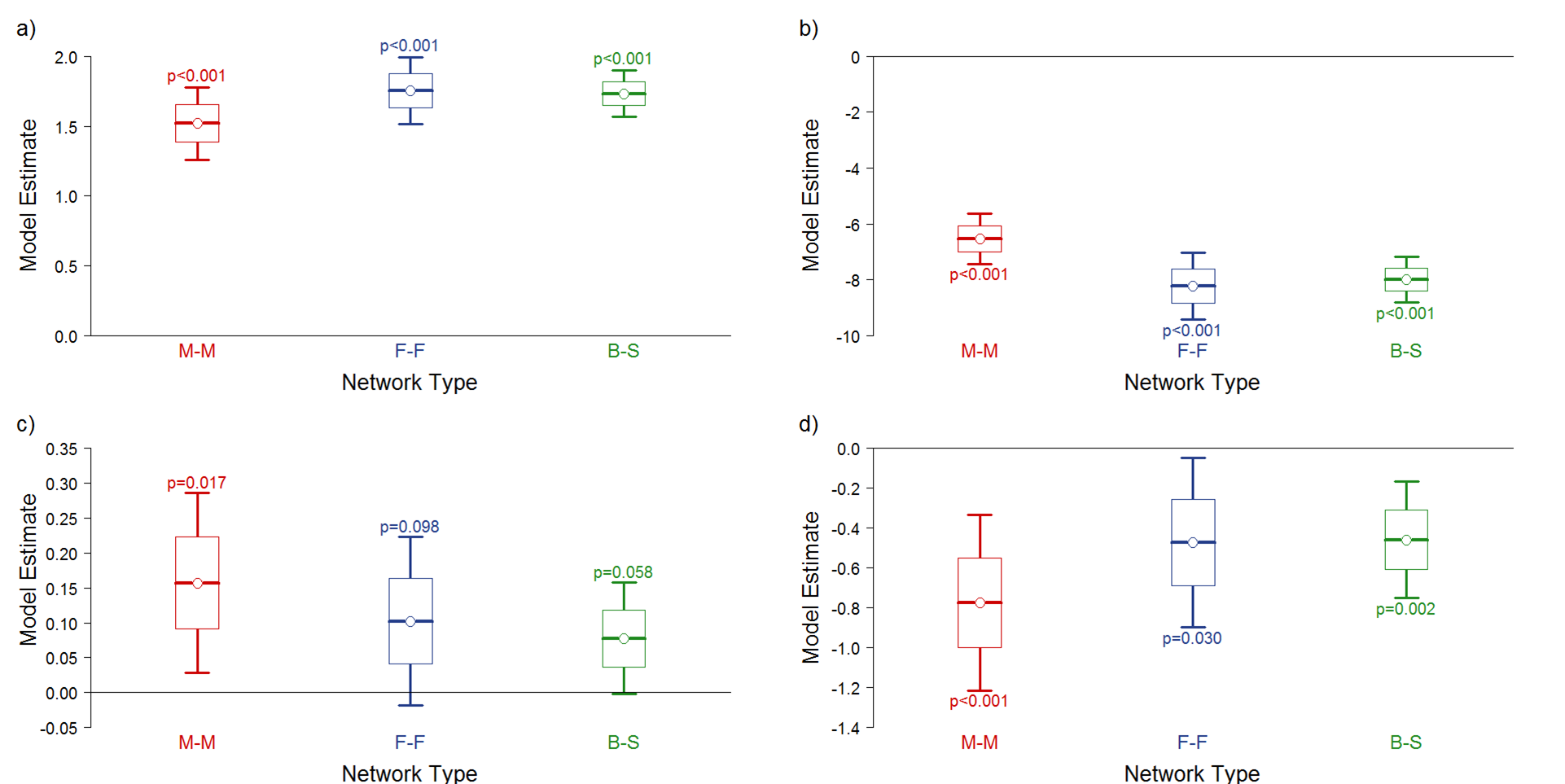


Figure S1. Model estimates from an exponential random graph model of between-group social contacts in badgers for a) the strength of contacts (sum term), b) the degree of contacts (non-zero term), c) the positive effect of TB infection status on strength (node factor term) and d) the effect of distance between main setts on strength (edge covariate term). Groups are defined using community assignment by a modularity algorithm. Results are shown for male-male networks (red), female-female networks (blue) and between-sex networks (green). Points are the model estimate, boxes are the standard error and bars are the 95% confidence intervals around this estimate. P values are for the difference between that estimate and 0.
